# Guideline from Japanese Society of Echocardiography: 2018 focused update incorporated into Guidance for the Management and Maintenance of Echocardiography Equipment

**DOI:** 10.1007/s12574-018-0370-z

**Published:** 2018-01-23

**Authors:** Masao Daimon, Makoto Akaishi, Toshihiko Asanuma, Shuji Hashimoto, Chisato Izumi, Shiro Iwanaga, Hiroya Kawai, Hiroyuki Toide, Akihiro Hayashida, Hirotsugu Yamada, Mitsushige Murata, Yutaka Hirano, Kengo Suzuki, Satoshi Nakatani, Committee for Guideline Writing, the Japanese Society of Echocardiography, M. Akaishi, C. Izumi, M. Daimon, M. Murata, Y. Hirano, K. Suzuki, H. Yamada

**Affiliations:** 1 Department of Clinical Laboratory, The University of Tokyo Hospital, 7-3-1 Hongo, Bunkyo-ku, Tokyo, 113-8655 Japan; 2 Tokai University Tokyo Hospital, Tokyo, Japan; 3 Osaka University Graduate School of Medicine, Osaka, Japan; 4 National Cerebral and Cardiovascular Center, Suita, Japan; 5 Tenri Hospital, Tenri, Japan; 6 Saitama Medical University, Moroyama, Japan; 7 Hyogo Brain and Heart Center, Himeji, Japan; 8 Gunma Cardiovascular Center, Maebashi, Japan; 9 The Sakakibara Heart Institute of Okayama, Okayama, Japan; 10 Tokushima University Hospital, Tokushima, Japan; 11 Keio University, Tokyo, Japan; 12 Kindai University, Osaka, Japan; 13 St. Mariana University School of Medicine, Kawasaki, Japan; 14 Osaka University Graduate School of Medicine, Osaka, Japan

**Keywords:** Echocardiography, Laboratory, Management, Maintenance, Guideline

## Abstract

Echocardiography plays a pivotal role as an imaging modality in the modern cardiology practice. Information derived from echocardiography is definitely helpful for a patient care. The Japanese Society of Echocardiography has promoted echocardiography for a routine clinical and research use. One of the missions of the Society is to provide information that is useful for high-quality examinations. To ensure it, we believe equipment in good conditions and a comfortable environment are important for both a patient and an examiner. Thus, the Committee for Guideline Writing, the Japanese Society of Echocardiography published brief guidance for the routine use of echocardiography equipment in 2015. Recently, the importance of international standardization has been emphasized in the medical laboratories. Accordingly, the committee has revised and updated our guidance for the routine use of echocardiography equipment.

## Introduction

Equipment in good conditions and a comfortable environment for both a patient and an examiner are essential for safe and high-quality echocardiography. Regular maintenance is indispensable to optimize the performance of the device. The Medical Service Law defines the maintenance requirements for medical devices including ultrasonography and the Japanese Circulation Society issues legal guidelines, but these guidelines are not specific to ultrasonography devices [1]. In addition to this, the Japanese Society of Sonographers has set out maintenance procedures and checklists for devices, but they are not specific to the echocardiograph [2]. In these circumstances, the Guideline Preparation Committee of the Japanese Society of Echocardiography published brief guidance for the routine use of echocardiographs [3].

Recently, medical laboratory has become required to follow the international standard. Echocardiographic laboratory plays important roles in the development of new drug and medical devices, or international clinical studies. Echocardiographic laboratory is also required to follow the international standard under the reliable quality control. Currently, ISO 15189 provided by International Organization for Standardization (ISO), is widely used for quality control of medical laboratory based on international standard. Furthermore, it has been recognized that echocardiographic laboratories can mediate health care-associated infection and echocardiographic laboratories need to take necessary measures of infection prevention and control. Based on these facts, here, we have revised our guideline.

Note: The present guidance omits any initial checks on the device at the time of purchase and regular inspections by the manufacturer, which should be carried out for each device according to the manufacturer’s recommendations. This guideline also makes no mention of continuous monitoring the patient safety which is taken precedence over everything else, because this guideline is just for maintenance of the devices or equipment.

## Maintenance supervisor for ultrasonographic devices

Although device checks should be carried out by all operators and doctors who operate echocardiographs, the Medical Services Law specifies the appointment of a maintenance supervisor. The maintenance supervisor is responsible for ensuring that the device is checked regularly and properly, and for resolving any problems when they occur. The supervisor is also responsible for proper enforcement of regular inspections by the manufacturer, including operational checks on connected devices and leakage current checks.

## Checklist

It is important to prepare a checklist for each device to enable multiple items to be checked efficiently in a short period. The date of inspection, items inspected, names of the inspector and supervisor, and any faults should be quickly and easily visible from the list. Preferably, items in the daily, weekly, and monthly inspections should be easy to evaluate from the list to make it easy to share information with the manufacturer’s maintenance engineer. The name of the manufacturer, the person responsible, and their contact number (sales, repair, night-time service center, etc.) and address should be listed for each device. A checklist that matches the circumstances at each facility should ideally be established referring to the examples in references [1–4].

## Carrying out inspections

1. Pre-operation inspection

Before operation, check the environment inside the examination room, the main ultrasonography device, and stocks and the arrangement of the supplies required.

A.Inspecting before turning the power on

1. Inspecting the conditions of use

Try to prepare the environment in the examination room from the viewpoint of the patient.

- Check that the ambient temperature and humidity in the examination room are comfortable. Check there are no traces of scent or perfume from the previous patient.

*Lie on the bed and check that there are no distractions or disturbances in the visible range including the ceiling, that the light is not directly in the patient’s line of sight, and that the downdraft from the air conditioner does not blow directly onto the patient.

- Check that the clothes basket, trash box, etc. near the bed are clean and placed neatly. Check that there is no dust, etc. on the floor.
- Check that the bed sheet, pillow, and towel are clean. Check that there is no dirt, hairs or dust on them and smooth out any creases.
- Check that the bed casters are locked. If the echo bed is an adjustable electric bed, check that it is returned to its original setting position.
- For transesophageal echocardiography and stress echocardiography or when examining a patient who is in an unstable condition, check the oxygen supply and suction tubes, and the emergency equipment including emergency medicines, injections, and Ambu bag. Check that the defibrillator/AED is ready to use.
- Inspecting the device surroundings
- Check that there is sufficient space around the device to avoid any buildup of heat.

*If the fan outlets (suction/exhaust ports) are in contact with the wall or curtains, the temperature inside the device may increase, causing it to fail.

- Check that the casters of the ultrasonic device are locked.
- Check that the power plug is fully inserted into the outlet.

*The main power plug for the ultrasonographic device must be connected directly to the white outlet for medical use (triple-pole outlet, AC 100 V, 15 A, 10 Ω or less grounding resistance, attached to a ground terminal). Connect the plug while the power to the main device is turned off.

*There are three types of power supplies in the hospital: white (ordinary supply), red (ordinary supply + private power generator backup), and green (ordinary supply + private power generator + battery backup). The red and green outlets are primarily for connecting important equipment such as life-support systems, so the ultrasonic device should be connected to the white outlet, not to the red or green outlets. When connecting the ultrasonographic device in a hospital ward, inform the section involved that you are connecting it and ensure it is the correct capacity before connecting. If there is no alternative to using an extension cord (triple-pole type), be careful to avoid a “star-connection” causing overloading circuit.

(3) Inspecting the ultrasonographic device

Inspecting the various cables

- Check that there are no tangled or twisted sections, or insulation breaks in the probe cable, power cable, or ECG monitor cable. Check the connections.
- Check that the LAN cable (for input/output of electronic patient records) is properly connected to the LAN outlet. Check for damage.

Inspecting the probe

- Check for any abnormalities such as damage or cracks to the acoustic lens surface or the handle.
- Check for any cracks, damage, or broken pins in the connector (connection with the main device).
- Check the transesophageal probe using the same procedure. Especially check for cracks, chipping, twisting, discoloration, etc. on the surface of the connector cable.

Inspecting the monitor screen

- Check that there is no dust, fingerprints, echo gel, etc. on the monitor screen.
- Inspecting the supplies
- Check that there is a good stock of recording paper, echo gel, disposable ECG electrodes.
- Inspecting the recording media
- When making video or DVD recordings, check that the record starting point is set to the finishing point of the previous examination and that there is enough recording space.
- When recording to a hard disk, check that there is sufficient capacity on the disk. (If there is not enough capacity on the hard disk, the behavior of the device may become unstable.) Backup the hard disk periodically onto media such as DVDs.

B.Inspecting after the power is turned on

1. Activating the device

After turning on the power supply, carry out an inspection with the room lights set to the same brightness as for the ECG test.

- Check that the device and peripheral equipment operate normally. Check that there are no error messages during operation. Check for any abnormal sounds.

*If an error message appears or any abnormal behavior occurs on the panel when turning on the device, write down or print out the error message and contact the manufacturer to get the device repaired.

- Check that the device is set to display the correct date.
- Check that indicators, switches and buttons on the control panel, trackball, and keyboard all operate normally.
- Check that the acoustic lens surface of the probe is not abnormally hot.
- Check that the transesophageal probe works normally when operating the angle knob. Check that the acoustic lens surface rotates smoothly.
- In a facility that is connected to the hospital information system or filing server, check that the work list and data transfer to the image server are available.
- Image quality of the monitor and peripheral equipment
- Check that the brightness and contrast are set correctly. Check the grey scale settings step-by-step from a black background to a white background.
- Check for any defects in the image or abnormal noise when setting the two-dimensional echo gain to a higher value.
- Check that the color tone changes as the color gain is increased.

*During inspection, turn down the color gain until clutter and noise disappear.

- Check the quality of the output image on the monitor screen.

*Check by printing out the image to the printer. If any noise appears, clean the print head.

*Output to video or DVD and compare the image with the monitor screen.

2. Daily inspection

At the end of each examination, carry out an inspection in preparation for the next examination. Parts of the pre-operation inspection should be repeated as follows.

∙Check that the ambient temperature and humidity in the examination room are comfortable. Check there are no traces of scent or perfume from the previous patient.

∙Check that the clothes basket, trash box, etc. near the bed are clean and placed neatly. Check that there is no dust, etc. on the floor.

∙Check that the bed sheet, pillow, and towel are clean. Check that there is no dirt, hairs or dust on them and smooth out any creases.

∙Check that the cables for the probe, ECG device, etc. are not tangled up.

∙Check that the bed casters are locked. If the echo bed is an adjustable electric bed, check that it is returned to its original setting position.

∙Check that no items have been left behind by the previous patient.

∙In transesophageal echocardiography, the patient may accidentally bite the probe during the examination. Check for any abnormalities in the appearance of the connecter cable during cleaning.

3. Inspection at the end of operation

Check the records to follow-up on any problems discovered during operation in preparation for the next patient. The pre-operation inspection before the next examination can be simplified by completing part of it at the end of the current examination.

∙Check for any gel adhering to the probe. Clean, sterilize, and disinfect the equipment according to the procedure described in the instruction manual.

∙Check that there is no dirt, gel, etc. on the control panel.

∙Check that all the images have been saved safely. In facilities that record to video or DVD, check the end point.

∙Replace any supplies that have been used.

4. Weekly inspection
  Clean the outside of the main device and probe holder according to the instruction manual.
  Check that the monitor stand attached to the device and that any peripheral equipment are firmly secured and not loose.
  Check the filters on the intake ports on the front/rear, side, and bottom of the device, and use a vacuum cleaner to clean any dust from the filters. Check that nothing is obstructing the exhaust port.
5. Monthly inspection
  Clean the main device and the printer and video recorder heads.
  Inspect and remove any dust, etc. from around the power outlet.
  In facilities that provide an emergency cart and medicine cabinet in the examination room, check the number of medicines in stock and their use-by dates, and refill or replace them as necessary.
6. Infection prevention and control (updated)

It should be recognized that echocardiographic laboratories can mediate health care-associated infection via contacts between patients and probe, electrode or sonographer [5]. For example, multi drug-resistant *Acinetobacter* *baumannii* can survive for a long time under the xeric environments and may cause health care-associated infection through these pathways. Therefore, sonographer should confirm patients’ information regarding infection of the resistant bacterium and take necessary measures of infection prevention and control according to the institutional rule before echocardiography, in addition to keeping laboratory clean regularly. Sonographers are required to wash their hands routinely before and after the examinations and wipe out the surface of echo-bed, probe, and electrode using a wet cloth with ethanol to achieve effective disinfection after the examination.

## Procedure for contacting the manufacturer’s call center

If a fault occurs in the device during use, contact the manufacturer promptly. The person in charge must act quickly to get the device repaired. To ensure this happens as smoothly as possible, in principle let an office administrative staff contact the manufacturer because there will be a maintenance contract and arrangements for paying repair charges between the hospital and the manufacturer.

Follow this procedure when contacting the call center:

1. Call the call center using the number on the label on the rear or side of the device, and give them the system number from the label. This will enable the manufacture to determine the hospital name, device type, and probes attached.
2. Give details of the fault including details of the operation being conducted when the problem happened. Also give details of any error messages.
3. Record the time the fault happened. This will allow the service engineer to check the error log.
4. When contacting the call center, record the date and time and the name of the person you spoke to.

## ISO15189 (updated)

International Organization for Standardization (ISO) is an independent, non-governmental international organization for providing specifications, guidelines or characteristics that can be used consistently over the world to ensure that materials, products, processes, and services are fit for their purpose. ISO 15189 is provided by ISO and is particular requirements for quality and competence specifies the quality management system requirements particular not only to echocardiography but to all medical laboratories. The first version of ISO15189 was published in 2003, it was revised in 2007 and the third edition of the standard was published in 2012 [6]. In Japan, the certification program for medical laboratories based on ISO15189, which was developed by Japan Accreditation Board (JAB) and Japanese Committee of Clinical Laboratory Standards (JCCLS), was launched in 2005 [7].

Recently, echocardiographic laboratories became often required to be certificated by ISO15189 for participating in international collaborative studies or clinical trials and the number of echocardiographic laboratories certificated by ISO15189 is increasing in Japan. To be a certificated laboratory, the echocardiographic laboratory has to be passed certification examination by JAB in Japan. The certification examination includes completion of “the quality management system” and “the competence of testing and calibration laboratories”. These are mainly examined with the accreditation scope applied by the facility. The points judged as incompatible in the examination need to be corrected and reported to JAB. Whether certification is possible or not is deliberated by the accreditation committee based on the content of the review and corrective action. The term of validity for the certification is 4 years, during which twice surveillance audits are conducted, and it is necessary to confirm the status that maintains the quality management system and competence of testing and calibration laboratories.
